# Development of a Novel Biosensor-Driven Mutation and Selection System via *in situ* Growth of *Corynebacterium crenatum* for the Production of L-Arginine

**DOI:** 10.3389/fbioe.2020.00175

**Published:** 2020-03-13

**Authors:** Meijuan Xu, Pingping Liu, Jiamin Chen, Anqi Peng, Taowei Yang, Xian Zhang, Zhenghong Xu, Zhiming Rao

**Affiliations:** ^1^Key Laboratory of Industrial Biotechnology, Ministry of Education, School of Biotechnology, Jiangnan University, Wuxi, China; ^2^Jiangnan University (Rugao) Food Biotechnology Research Institute, Rugao, China

**Keywords:** biosensor, transcription factors, high-throughput screening, L-arginine, ArgR, *sacB*, *Corynebacterium crenatum*

## Abstract

The high yield mutants require a high-throughput screening method to obtain them quickly. Here, we developed an L-arginine biosensor (ARG-Select) to obtain increased L-arginine producers among a large number of mutant strains. This biosensor was constructed by ArgR protein and *argC* promoter, and could provide the strain with the output of bacterial growth via the reporter gene *sacB*; strains with high L-arginine production could survive in 10% sucrose screening. To extend the screening limitation of 10% sucrose, the sensitivity of ArgR protein to L-arginine was decreased. *Corynebacterium crenatum* SYPA5-5 and its systems pathway engineered strain Cc6 were chosen as the original strains. This biosensor was employed, and L-arginine hyperproducing mutants were screened. Finally, the HArg1 and DArg36 mutants of *C. crenatum* SYPA5-5 and Cc6 could produce 56.7 and 95.5 g L^–1^ of L-arginine, respectively, which represent increases of 35.0 and 13.5%. These results demonstrate that the transcription factor-based biosensor could be applied in high yield strains selection as an effective high-throughput screening method.

## Introduction

Microbial production has become a dominant method to generate various substances, such as amino acids (Wendisch, 2014), organic acids (Alonso et al., 2015), and vitamins (Ami et al., 2013). To obtain a large number of value-added compounds, the conventional mutagenesis strategy (Yakovleva et al., 2008; Sun et al., 2013) has been regarded as a desired approach in the past few years, however, it is hard to obtain the hyperproducing strains among a large number of mutant strains. Recently, advances in the metabolic engineering strategies (Kubicek et al., 2009) have enabled rationally designed microorganisms, and considerable attention has been focused on the modification of enzymes in metabolic pathways (Schneider et al., 2011). In one such example, based on the *Corynebacterium glutamicum* AR1 strain, removal of the regulatory repressors of the arginine operon, optimization of the NADPH level, and disruption of the L-glutamate exporter allowed the strain to produce 92.5 g L^–1^ of L-arginine in a 5-L bioreactor (Park et al., 2014). This has led to successful overproduction of quantities of target compounds. However, because of the widely used of this approach, the L-arginine production of strains has reached a bottleneck. Therefore, random mutagenesis is still an effective strategy for improving metabolites production. To overcome the defects of conventional screening methods, biosensors are routinely employed to detect the metabolites production and obtain the hyperproducing strains in a short time. This is mainly achieved by a transcription factor (TF)-based system with the output of reporter genes, including fluorescence intensity or antibiotic resistance (Mahr et al., 2015; Liu et al., 2017).

Naturally, microorganisms have their own regulatory system to maintain the metabolic balance. Based on the characteristics of the binding of a protein to a molecule and the variability of protein conformation, the function and activity can be used of controlling the expression of downstream genes (Liu et al., 1998). The TF is a good example, exhibiting specific binding to target metabolites and up- or down-regulation of gene expression by changing structure. The LysG TF in the LysR family of *C. glutamicum* is a typical example of high-throughput screening. Based on the LysG TF, which can sense L-arginine, L-lysine, and L-histidine and interact with the corresponding promoter of *lysE*, the biosensor of pSenLys-Spc has been successfully constructed in *C. glutamicum* (Schendzielorz et al., 2014). In addition, the regulatory protein FadR, which is responsive to acyl-CoA, has been employed for a dynamic control of biosynthetic pathways (Zhang et al., 2012). A QdoR-based biosensor was also applied to monitor kaempferol production in single cells by flow cytometry (Siedler et al., 2014). Inspired by these features, TFs can be developed as the main elements to construct whole-cell biosensors that can regulate the transcription level of reporter genes in response to specific metabolites (Scognamiglio et al., 2015).

L-Arginine production in *C. glutamicum* is organized by an *arg* cluster of *argCJBDFRGH* that can be classified into *argCJBDFR* and *argGH* operons. The two operons regulate most enzymes that convert L-glutamate into L-arginine, and then, transcription is initiated by their respective *argC* and *argG* promoters. Furthermore, ArgR is considered a negative regulator that represses the transcription of *argCJBDFR* in response to high L-arginine concentration and recognizes its binding motifs on the *argC* promoter (Chen et al., 2014). The *argC* promoter is at the first position of the *arg* cluster and can influence the expression of downstream genes (Yim et al., 2011). However, the ArgR repressor cannot associate with the *argG* promoter because it is not responsible for *argGH* (Theron and Reid, 2011). Some strategies have been used to improve L-arginine production in *Corynebacterium crenatum* SYPA5-5 (Xu et al., 2011a; Guo et al., 2017). An engineered strain, Cc6, was constructed, which involved removal of regulatory repressors of the L-arginine operon, optimization of the NADPH level, disruption of the L-glutamate exporter, and flux optimization of rate-limiting L-arginine biosynthetic reactions (Man et al., 2016).

In this study, we attempted to utilize ArgR protein and the corresponding promoter of *argC* to construct the ARG-Select biosensor. This biosensor was applied in *C. crenatum* SYPA5-5 and its engineered strain Cc6. Because of the lethal effects of *sacB* in the presence of 10% sucrose (Gay et al., 1983; Pelicic et al., 1996), it was chosen as the reporter gene to reflect the intracellular L-arginine concentration. This biosensor provides a high-throughput screening method based on the output of cell growth. Meanwhile, the fluorescence intensity of *gfp* was also used to monitor the change in L-arginine production in cells, which makes intracellular L-arginine concentration visible, and the accuracy of the biosensor system was demonstrated. Finally, both the L-arginine production of the two strains was increased, indicating that the application of the ARG-Select biosensor is an efficient method to obtain desired mutants in good yields.

## Materials and Methods

### Strains and Plasmids

All the strains, plasmids and their sources are listed in Table 1. The strain of *C. crenatum* SYPA5-5 (CGMCC No. 0890) was obtained by UV and EMS mutagenesis, and it could produce L-arginine (30 g L^–1^) in shake-flask fermentation under optimal culture conditions (Xu et al., 2013). The Cc6 strain was obtained by system pathway engineering from *C. crenatum* SYPA5-5, and its L-arginine production could reach 87.3 g L^–1^ in fed-batch fermentation (Man et al., 2016). The *C. glutamicum*/*E. coli* shuttle vector of pDXW-10 was used for *C. glutamicum* expression (Xu et al., 2010). The reporter gene of *sacB* encodes the *Bacillus subtilis* levansucrase, and *sacB* expression is lethal to strains in the presence of 10% sucrose (Pelicic et al., 1996).

**TABLE 1:** Strains and plasmids used in this study.

**Strains/plasmids**	**Characteristics**	**Resource**
**Strains**		
*E. coli*		
JM109	A model wild type *E. coli*	Lab stored
*EcoPC-sacB*	*E. coli* JM109 with *PC*-*sacB* plasmid	This work
***C. glutamicum***		
ATCC 13032	A model wild type *C. glutamicum*	Lab stored
*Cg*Δ*argR*	*C. glutamicum* ATCC 13032 with deletion of *argR* gene	Lab stored
*CgPC-sacB*	*C. glutamicum* ATCC 13032 with *PC*-*sacB* plasmid	This work
***C. crenatum***		
SYPA5-5	A model wild type *C. crenatum*	Lab stored
Cc6	An L-arginine high-producing *C. crenatum* SYPA5-5 with multis metabolic engineering methods	Lab stored (Man et al., 2016)
*CcPC-sacB*	*C. crenatum* SYPA5-5 with *PC*-*sacB* plasmid	This work
**Plasmids**		
pDXW-10	*E. coli*–*C. glutamicum* shuttle vector, Km^r^ or Amp^r^	Lab stored (Xu et al., 2010)
*PC-sacB*	*argC* promoter linked with *sacB* by overlap extension PCR and ligated on plasmid pDXW-10, Km^r^ or Amp^r^	This work
*PC-gfp*	*argC* promoter linked with *gfp* by overlap extension PCR and ligated on plasmid pDXW-10, Km^r^ or Amp^r^	This work
pSenArg-*sacB*	*argR* gene ligated on plasmid *PC-sacB*, Km^r^ or Amp^r^	This work
pSenArg-*gfp*	*argR* gene ligated on plasmid *PC-gfp*, Km^r^ or Amp^r^	This work

### The ARG-Select Biosensor Plasmid Construction

All of the primers and restriction enzymes are listed in Table 2. Primers were synthesized by Genewiz (Suzhou, China). The restriction enzymes, DNA polymerase of PrimeSTAR HS, and T4 DNA ligase were purchased from TaKaRa (Dalian, China). Using genomic DNA from *C. glutamicum* ATCC 13032 as a template, the gene encoding ArgR was amplified with *argR*-F and *argR*-R primers. The *sacB* gene was amplified from the pK18*mobsacB* plasmid with *sacB*-F and *sacB*-R primers, and the *gfp* gene was obtained by PCR from the pDXW-10*gfp* plasmid using the *gfp*-F and *gfp*-R primers. The native promoter of *tac-M* was replaced by the P*_*lac*_* promoter at the *Bam*HI and *Eco*RI sites, and the P*_*lac*_* promoter was amplified with the P*_*lac*_*-F and P*_*lac*_*-R primers. The *argC* promoter was linked with *sacB* and *gfp* by overlap extension PCR and digested with *Hin*dIII, *Afl*II, *Pst*I, and *Bgl*II, and the PCR products were ligated into the pDXW-10 plasmid. Briefly, to construct the biosensor plasmids of pSenArg-*sacB* and pSenArg-*gfp*, the *argR* gene was ligated into the *PC*-*sacB* and *PC*-*gfp* plasmids using *Eco*RI and *Not*I. Finally, the recombinant plasmids were transformed into *C. glutamicum* ATCC 13032 and *C. crenatum* SYPA5-5.

**TABLE 2:** Primers used in this study.

**Primers**	**Sequences**	**Restriction enzymes**
*PC*-*sacB*-F1	CCC**AAGCTT**AAATTCATG CTTTTACCCACTTGC	*Hin*dIII site
*PC*-*sacB*-R1	GCAAACTTTTTGATGTTCA TAGTTACACCATACACG	Fusion overlap
*PC*-*sacB*-F2	CGTGTATGGTGTAACTAT GAACATCAAAAAGTTTG	Fusion overlap
*PC*-*sacB*-R2	CCC**ACATGT**TTATT TGTTAACTGTTAATTGTCC	*Afl*II site
*argR*-F	CG**GAATTC**ATGTCCCTTG GCTCAACCCC	*Eco*RI site
*argR*-R	ATTT**GCGGCCGC**TTAAG TGGTGCGCCCGCTGAG	*Not*I site
P*_*lac*_*-F	CG**GGATCC**TAATGGA TTTCCTTACG	*Bam*HI site
P*_*lac*_*-R	CG**GAATTC**ATAATAA CCGGGCAGGCC	*Eco*RI site
*PC*-*gfp*-F1	GA**AGATCT**AAAT TCATGCTTTTACCCACTTGC	*Bgl*II site
*PC*-*gfp*-R1	GTTCTTCTCCCTTAC CCATAGTTACA CCATACACGTTATGCATG	Fusion overlap
*PC*-*gfp*-F2	CATGCATAACGTGTATG GTGTAACTATG GGTAAGGGAGAAGAAC	Fusion overlap
*PC*-*gfp*-R2	AA**CTGCAG**TTAT TTGTATAGTTCATCCATG	*Pst*I site

*Bold sequences highlight recognition sites for restriction enzymes.*

### Quantitative Real-Time PCR Analysis

Strains were cultivated at 30°C and 180 rpm and induced by 0.05 mM IPTG when the OD_600_ reached 0.5. Cells were harvested by centrifugation at 12,000 rpm, at 4°C for 2 min, suspended in 100 μL TE buffer containing 20 μL lysozyme (150 mg L^–1^) and incubated for 30 min. Total RNA was purified using a MasterPure^TM^ RNA purification kit (Vazyme, China). The first strand cDNAs were synthesized by HiScript^®^II Q RT SuperMix (Vazyme, China) using total RNA (0.5 μg). For RT-qPCR, the 16S rRNA was chosen as the endogenous control. The reaction mixture was prepared in a qPCR tube and consisted of 10 μL of 2× ChamQ Universal SYBR qPCR Master Mix, 0.4 μL of forward and reverse primers (10 μM each) respectively, 1.0 μL of cDNA, and 8.2 μL of ddH_2_O. The RT-qPCR was performed on a Bio-Rad CFX96 Manager PCR system (Bio-Rad, United States) using the following parameters: 95°C for 30 s, followed by 40 cycles of denaturation at 95°C for 10 s and 60°C for 30 s and extension at 72°C for 20 s. The 2^–ΔΔ*Ct*^ method was applied to analyze the data, which were normalized to the transcription level of 16S rRNA.

### Medium and Growth Conditions

Both *Corynebacterium* sp. strains were first cultivated at 30°C in broth medium (BHI) with 50 μg mL^–1^ of kanamycin or ampicillin added. The preliminary screening agar medium was L-arginine production fermentation medium (glucose 25 g L^–1^, yeast extract 10 g L^–1^, (NH_4_)_2_SO_4_ 40 g L^–1^, KH_2_PO_4_ 1.5 g L^–1^, KCl 1 g L^–1^, MgSO_4_.7H_2_O 0.5 g L^–1^, FeSO_4_.7H_2_O 0.02 g L^–1^, and MnSO_4_.H_2_O 0.02 g L^–1^) containing 10% sucrose and 0.05 mM IPTG. Three days later, the mutant colonies were inoculated into 24-deep-well plates with 3 mL of fermentation medium without sucrose, and they were cultivated for 48 h until the cell’s concentration reached an OD_600_ = 3.5–4.0. For shake-flask fermentation, mutant strains were activated on fermentation agar plates for 24 h and inoculated in 10 mL of seed medium (BHI). Then, 1.5 mL of seed culture was transferred into 30 mL of fermentation medium (glucose 150 g L^–1^, yeast extract 10 g L^–1^, (NH_4_)_2_SO_4_ 40 g L^–1^, KH_2_PO_4_ 1.5 g L^–1^, KCl 1 g L^–1^, MgSO_4_.7H_2_O 0.5 g L^–1^, FeSO_4_.7H_2_O 0.02 g L^–1^, MnSO_4_.H_2_O 0.02 g L^–1^, and CaCO_3_ 20 g L^–1^) in 250-mL shake flasks and cultivated at 220 rpm for 96 h. Subsequently, the yield of L-arginine was measured. Repeated fermentation of mutant strains was required to ensure genetic stability.

### ARTP Mutagenesis

Before treating with atmospheric and room temperature plasma (ARTP) mutagenesis, cells were cultivated to the mid and late stages of logarithmic growth. The cells were washed twice with sterile saline and diluted to an OD_600_ = 0.2–0.3. Mutagenesis was performed using the ARTP mutation breeding system (Si Qing Yuan Biotechnology Co., Ltd., China). First, 10 μL of cell culture was placed on a piece of metal slide and put on the minidisc. The main parameters included the input power (100 W), ventilation flow rate (10 L min^–1^), and the distance between the sample plate and ion generator (2 mm).

Cells were treated by an ion beam for 30 s. Then, the sheet iron with 10 μL of mutagenized cells was placed into 990 μL of sterile saline. Finally, the cells were diluted and spread on the fermentation medium plates containing 10% sucrose. After incubation at 30°C for 3 days, the numbers of control colonies (A) and surviving colonies (B) were counted and the lethality rates were calculated with the following equation: (A − B)/A × 100%.

### Flow Cytometry

Before cells were analyzed by flow cytometry, 10 μL of mutagenized cells with the reporter gene of *gfp* were cultivated for 6 h in 1.0 mL of fermentation medium containing 50 μg mL^–1^ of kanamycin or ampicillin for cells’ recovery. Afterward, 100 μL of the cell culture was incubated in 10 mL of fresh medium and induced by IPTG until the OD_600_ reached 0.5. Then, the cells were washed twice with phosphate buffer (pH 7.4) and diluted to OD_600_ = 0.1.

Fluorescence sorting of cells was performed by a FACSAria II flow cytometer (Becton Dickinson, San Jose, United States) using an excitation wavelength of 488 ± 20 nm and an emission wavelength of 520 ± 20 nm at a sample pressure of 70 psi. The forward-scatter characteristics (FSC) and side-scatter characteristics (SSC) were detected with a 488-nm laser. Non-fluorescent cells of *C. crenatum* SYPA5-5 cells were used to exclude the background for cell sorting. Two gates with different fluorescence intensity were circled out based on pre-analysis of mutagenized cells, and four-way purity was used as the precision mode for cell sorting with a threshold rate of up to 6,000 events s^–1^. Data were analyzed using FlowJo v10.0.7 analysis software (Tree Star, Ashland, United States).

### Fed-Batch Fermentation

Fed-batch fermentation was carried out in 5-L fermenters (BIOTECH-5BG, Baoxing Co., China). Strains were first activated on an agar plate (glucose 30 g L^–1^, NaCl 10 g L^–1^, peptone 10 g L^–1^, yeast extract 5 g L^–1^, agar 20 g L^–1^, corn steep liquor 25 g L^–1^, pH 7.0) for 24 h. Single colonies were inoculated into seed medium (glucose 40 g L^–1^, corn steep liquor 50 g L^–1^, K_2_HPO_4_ 1.5 g L^–1^, MgSO_4_.7H_2_O 0.5 g L^–1^, CaCO_3_ 1 g L^–1^, urea 1 g L^–1^, (NH_4_)_2_SO_4_ 10 g L^–1^, pH 7.0) and cultivated at 30°C and 180 rpm for 24 h until the OD_600_ = 16–18. The seed culture was then transferred into 1.5 L of fermentation medium (glucose 40 g L^–1^, corn steep liquor 50 g L^–1^, (NH_4_)_2_SO_4_ 30 g L^–1^, KH_2_PO_4_ 2 g L^–1^, MgSO_4_.7H_2_O 0.5 g L^–1^, urea 1 g L^–1^, molasses 10 g L^–1^, pH 7.0) at 1:100 vol/vol. The feed medium (corn steep liquor 120 g L^–1^, molasses 120 g L^–1^, KH_2_PO_4_ 12 g L^–1^, MgSO_4_.7H_2_O 3 g L^–1^, pH 7.0) was fed into the fermenters when the concentration of glucose was less than 20 g L^–1^. The pH was controlled at 7.0 by 50% NH_3_.H_2_O, and the agitation speed was 600 rpm.

### Analytical Methods of L-Arginine

The production of L-arginine in 24-deep-well plates was measured by a modified Sakaguchi reagent spectrophotometric method (Messineo, 1966; Mitić et al., 2013). This method depends on the guanidyl on L-arginine, which can produce a purple substance with a mixture of naphthol and diacetyl in an alkaline medium. The reaction mixture consisted of 1 mL of sample solution, 4 mL of 0.375 M NaOH and 1 mL of color developing agent (5 g of 1-naphthol and 2.5 mL of 1% diacetyl dissolved in 100 mL normal propyl alcohol). The reaction was incubated at 30°C for 30 min, and the absorbance was measured at 520 nm.

For the shake-flask and fed-batch fermentation, cell culture was centrifuged at 8,000 rpm for 1 min, and 1 mL of culture supernatant was treated with 1 mL of 0.5 mol L^–1^ NaHCO_3_ and 150 μL of 1% DNFB-acetonitrile. Then, the mixture was heated at 60°C in darkness for 60 min. L-Arginine production was detected by using an HPLC system, and the separation was achieved by using a platisil 5 μm ODS, 250 × 4.6 mm C18 column. The mobile phase consisted of 0.1 mol L^–1^ NaAc (pH 6.4)-acetonitrile (86:14), with a flow rate of 1.0 mL min^–1^, injection volume of 10 μL, column temperature of 30°C, and detection wavelength of 360 nm.

### Structure Modeling

Homology modeling of the ArgR protein was carried out with SWISS-MODEL^1^ using the structure of *Mycobacterium tuberculosis* (PDB: 6A2Q) as a template. The binding of the L-arginine molecule to the ArgR protein was included in the structure. The model was assembled primarily as a tool to predict mutation sites. Model observation, image processing, and correlation analyses regarding the binding pocket of ArgR protein were performed by PyMOL program (Grell et al., 2006; Hagelueken et al., 2012).

## Results

### Design and Construction of the ARG-Select Biosensor

We proposed to improve the L-arginine production in *C. crenatum* SYPA5-5 and its system metabolic engineered strain Cc6 by constructing an L-arginine biosensor. Referring to the reported pSenLys-Spc biosensor system (Schendzielorz et al., 2014), we first attempted to construct a similar system in *C. crenatum* SYPA5-5 to obtain hyper-arginine producers. Indeed, the L-Lys/L-Arg exporter LysG could be transported to L-lysine and L-arginine, and it was regulated by the LysR regulator. In *C. crenatum* SYPA5-5, the pSenLys-Spc biosensor did not respond to the high L-arginine concentration as well as expected. Due to this finding, we speculated that an important regulator of ArgR was involved in the L-arginine synthesis in *C. glutamicum*.

**FIGURE 1:**
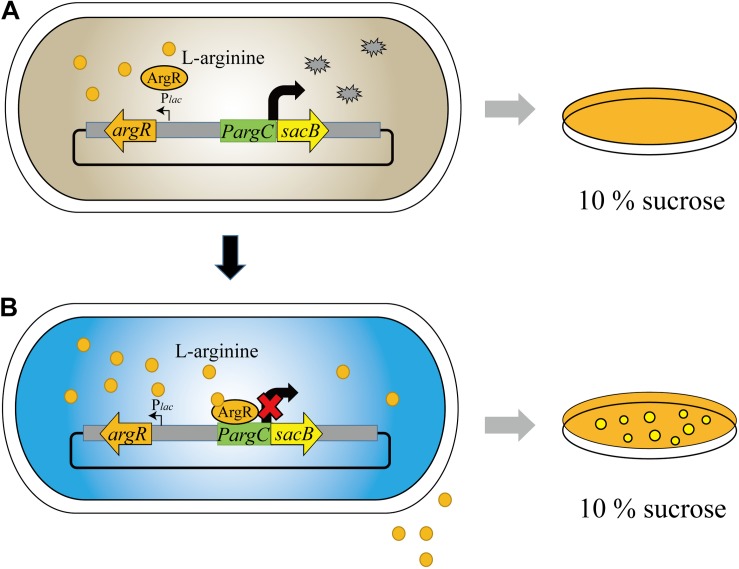
Design and construction of the ARG-Select biosensor. **(A)** No cells grew on 10% sucrose plate at low L-arginine concentration. **(B)** Cells grew on 10% sucrose plate with increased L-arginine concentration.

Our biosensor system utilized the ArgR protein from *C. glutamicum* to downregulate the *argC* promoter in response to high L-arginine concentration, which can be reflected by the reporter gene of *sacB*. Hence, the cell can only survive on 10% sucrose when the L-arginine concentration reaches a high value, and we named the biosensor as “ARG-Select” (Figure 1). The gene encoding ArgR was driven by the inducible P*_*lac*_* promoter, and the expression of the reporter gene was controlled by the *argC* promoter. The P*_*lac*_* promoter was induced by IPTG in the preliminary screening plate containing 10% sucrose, and L-arginine production was measured in the fermentation medium without IPTG added. Moreover, to demonstrate the effectiveness of this system, the *gfp* gene was also employed to monitor the L-arginine concentration by fluorescence intensity. Based on the results, the L-arginine concentration can be converted into cell survival and fluorescence signals of the ARG-Select biosensor.

### Sensitivity of the ARG-Select Biosensor to Extracellular L-Arginine

**FIGURE 2:**
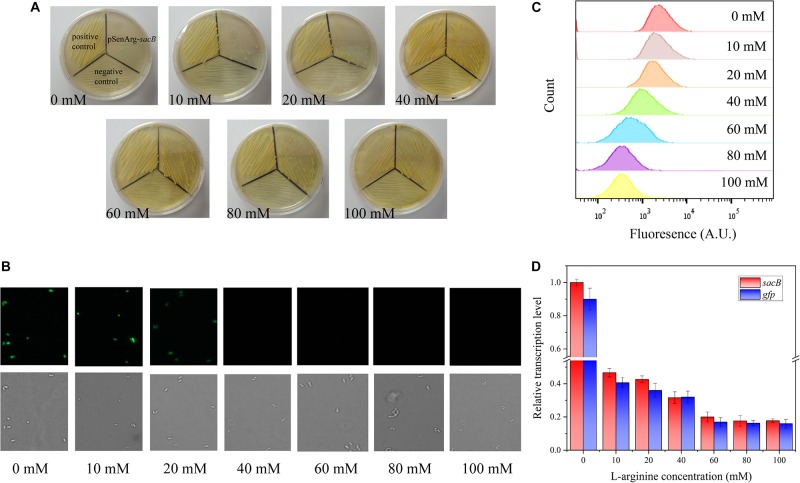
**(A)** Growth performance of strains on 10% sucrose plates at different L-arginine concentrations. From left to right: 0, 10, 20, 40, 60, 80, and 100 mM L-arginine. The positive control (harboring an empty plasmid) is shown in the top left corner, the negative control (harboring the *PC-sacB* plasmid) is shown at the bottom, and the strain carrying the pSenArg-*sacB* plasmid is shown in the top right corner. The growth performance of strains improved as L-arginine concentration increased. **(B)** Fluorescence microscope images of the strain carried the pSenArg-*gfp* plasmid with 0, 10, 20, 40, 60, 80, and 100 mM L-arginine concentrations. **(C)** FACS analysis of the fluorescence of strain carried pSenArg-*gfp* plasmid at different L-arginine concentration. **(D)** Relative transcription level of the *sacB* and *gfp* reporter genes at different L-arginine concentrations.

To evaluate the feasibility of the ARG-Select biosensor, the cell response to the L-arginine concentration was investigated. Different L-arginine concentrations ranging from 0 to 100 mM were added to the BHI medium plate containing 0.05 mM IPTG and 10% sucrose. Strains were cultivated on these plates by streaking inoculation, and the growth performance was observed after 3 days. A different growth status was observed on the increased L-arginine concentration plates (Figure 2A). No surviving cells were found on the 0 mM L-arginine plate, and only a few cells could grow when 10 mM L-arginine was added. A clear increase in cell growth was noted when the L-arginine concentration reached 60 mM. Complete growth of cells was observed at L-arginine concentrations of 60, 80, and 100 mM. For the *gfp*-based ARG-Select biosensor, the fluorescence was decreased with increased L-arginine concentrations (Figures 2B,C), and cells’ fluorescence was undetectable at 40 mM L-arginine.

Finally, RT-qPCR was performed on the recombinant ARG-Select biosensor to analyze the change in reporter gene expression at different L-arginine concentrations. The expression levels of *sacB* and *gfp* were decreased by 2.13- and 2.38-fold at 10 mM L-arginine and 5.02- and 5.88-fold at 60 mM L-arginine (Figure 2D). However, at 80 and 100 mM L-arginine, the strains exhibited the maximum growth, and the fluorescence intensity decreased to the lowest level. These results demonstrate a correlation between the cell growth or fluorescence and L-arginine concentration (0–60 mM), and the ARG-Select biosensor system exhibits a sensitive response to different L-arginine concentrations.

### Verification of ARG-Select Biosensor Based on Reporter Gene of *gfp*

**FIGURE 3:**
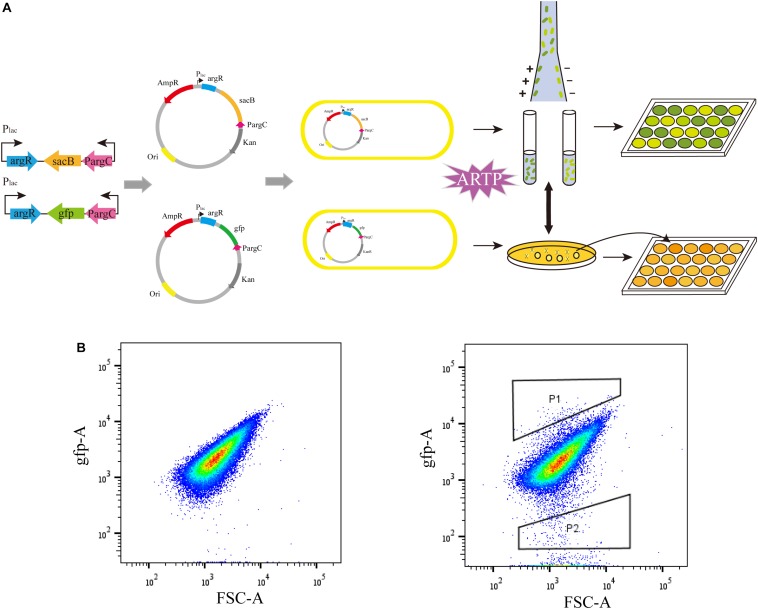
**(A)** The process of ARG-Select biosensor construction and L-arginine hyperproducing strains’ selection. **(B)** FACS analysis of the strain that carried the pSenArg-*gfp* plasmid. The non-mutagenized parent strain is on the left, and the mutagenized strain is on the right. Cells with strong fluorescence are in gate P1, and cells with weak fluorescence are in gate P2.

To examine the effectiveness of the ARG-Select biosensor, the *gfp*-based biosensor was used to further verify the relationship between fluorescence intensity and L-arginine production (Figure 3). The strain that carried the pSenArg-*gfp* plasmid was treated by ARTP mutagenesis and then analyzed by FACS. The mutagenized cells were sorted into two gates depending on whether the fluorescence was higher (P1) or lower (P2) than the control. Subsequently, cells in the two gates were collected and spread on agar plate with 50 μg mL^–1^ of kanamycin or ampicillin added. We randomly selected 200 strains from each gate and determined the L-arginine production in 24-deep-well plates for 48 h. Cells in P1 showed strong fluorescence and could produce 1.91 g L^–1^ of L-arginine on average; the L-arginine production of cells in P2 showed low fluorescence, and the cells could produce 2.48 g L^–1^ of L-arginine. The difference of L-arginine production between P1 and P2 cells was statistically significant (*P* < 0.01). Most of the weak cells could not be further identified and isolated by FACS. This result indicated that the fluorescence in a *gfp*-based biosensor system was capable of predicting the L-arginine yield of strains. The obvious difference of the L-arginine production between the strains in the two gates demonstrated the ability of the ARG-Select biosensor.

### The Applicability of the ARG-Select Biosensor System

**FIGURE 4:**
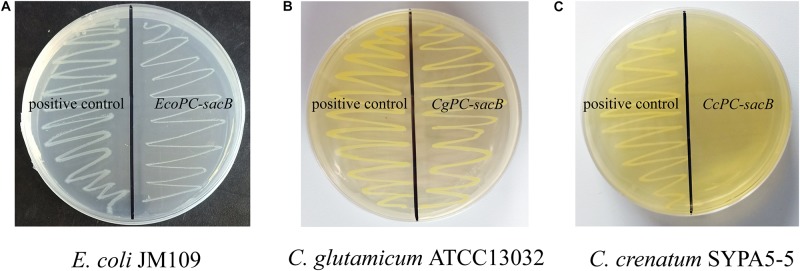
Growth performance of different strains on agar plates containing 10% sucrose. **(A)** *E. coli*, **(B)** *C. glutamicum* ATCC 13032, and **(C)** *C. crenatum* SYPA5-5. The positive control carrying an empty plasmid is on the left, and the strain carrying the *PC-sacB* plasmid is on the right.

*Corynebacterium crenatum* SYPA5-5 is a mutant strain that could produce 30 g L^–1^ L-arginine in our study. Previously, it was reported that a negative mutation (C → T) of *argR* occurred in *C. crenatum* SYPA5-5 that led to obviously increased expression of the *argC*–*argH* cluster (Xu et al., 2013). To investigate the ArgR background expression of the ARG-Select biosensor in different strains, the *PC-sacB* plasmid was constructed, and strains were inoculated on plates containing 10% sucrose. As shown in Figure 4A, the expression of *sacB* was first verified in *E. coli*, and we found that the cell growth was not significantly inhibited. This result indicated that weak expression of *sacB* occurred because the *argC* promoter was not suitable for *E. coli*. Next, the recombinant plasmid was transformed into *C. crenatum* SYPA5-5 and *C. glutamicum* ATCC 13032. As shown in Figures 4B,C, the ARG-Select biosensor in *C. glutamicum* ATCC 13032 with functional ArgR did not cause lethality in cells, whereas no *C. crenatum* SYPA5-5 cells could survive under 10% sucrose pressure. Therefore, the intracellular ArgR could affect cell lethality with *sacB* expression via the regulation of the ARG-Select biosensor. Subsequently, the *PC-sacB* plasmid was transformed into the recombinant strain of *C. glutamicum* ATCC 13032/Δ*argR*, and *sacB* expression led to the complete lethality of cells. The above results showed that our ARG-Select biosensor system could work well in *C. crenatum* SYPA5-5 for the inactivation of *argR*. However, when this ARG-Select biosensor was applied to other *Corynebacterium* species, the deprivation of the background *argR* gene in the strain eliminated the original repression of *sacB* under the *argC* promoter.

### Relationship Between Cell Growth and L-Arginine Production

**FIGURE 5:**
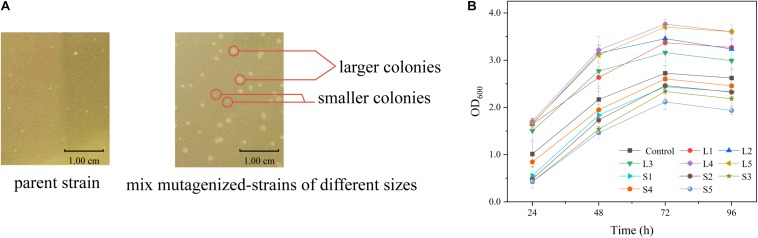
**(A)** Colony sizes of strains carrying the pSenArg-*sacB* plasmid on fermentation medium plates containing 10% sucrose. The left side shows a non-mutagenized parent strain, and the right side shows mixed mutagenized strains including high L-arginine producing strains, medium L-arginine producing strains, and low L-arginine producing strains. **(B)** Growth curves of large and small mutagenized strains. The black square line is the control parent strain. Strains L1, L2, L3, L4, and L5 are large strains, and S1, S2, S3, S4, and S5 are small strains.

The relationship between cell growth and L-arginine production was further verified in the *sacB*-based ARG-Select biosensor. The strain was mutagenized by ARTP and then cultivated on preliminary screening plates containing IPTG and 10% sucrose. A total of 372 large colonies (colony diameter ≥1.20 mm) grew on the plate, and they were randomly picked with 200 small colonies (colony diameter ≤0.80 mm) to determine the L-arginine production in 24-deep-well plates. An obvious difference in L-arginine production was found between large and small colonies (Figure 5A). Most of the small colonies could only produce 1.85–2.40 g L^–1^ of L-arginine, and they could not produce more than 2.45 g L^–1^ of L-arginine, whereas most of the large colonies produced 2.55–2.79 g L^–1^ of L-arginine, and they could not produce less than 2.37 g L^–1^. This result demonstrated that the L-arginine yield of large colonies was higher than that of small colonies. Moreover, five large (L1, L2, L3, L4, and L5) and five small (S1, S2, S3, S4, and S5) colonies were cultivated to obtain the growth curves (Figure 5B); the cell density (OD_600_) was measured every 24 h. As expected, different growth rates were shown between the large and small colonies, and strains with high L-arginine production showed a fast growth rate (Table 3). Therefore, it can be inferred that L-arginine production is closely related to cell growth in the ARG-Select biosensor. Instead of sophisticated analytical methods such as HPLC or chemical coloring, the mutagenized strains with high L-arginine yield can simply be selected by cell growth, which greatly simplifies the operation methods and steps.

**TABLE 3:** Maximum growth rate and L-arginine yield of the 10 large and small mutagenized strains.

**Strains**	**Control**	**L1**	**L2**	**L3**	**L4**	**L5**	**S1**	**S2**	**S3**	**S4**	**S5**
Maximum growth rate (h^–1^)	0.031 ± 0.002	0.043 ± 0.004	0.044 ± 0.002	0.039 ± 0.003	0.050 ± 0.002	0.049 ± 0.003	0.026 ± 0.002	0.026 ± 0.001	0.023 ± 0.001	0.029 ± 0.003	0.020 ± 0.004
L-Arginine (g/L)	2.44 ± 0.11	2.67 ± 0.12	2.78 ± 0.13	2.56 ± 0.13	2.89 ± 0.14	2.88 ± 0.12	1.85 ± 0.12	1.88 ± 0.10	1.75 ± 0.13	2.28 ± 0.12	1.59 ± 0.12

### Optimization of the Sensitivity of the ArgR Regulator to L-Arginine

**FIGURE 6:**
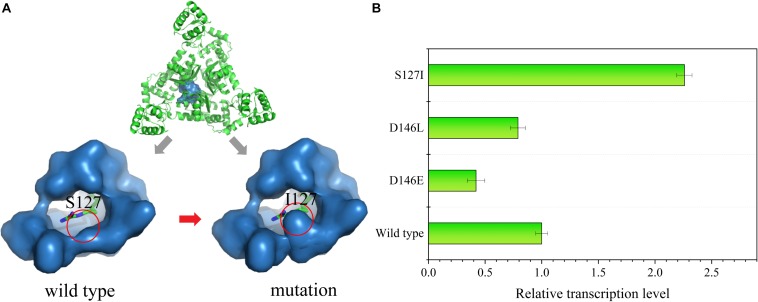
**(A)** Structural characterization of the ArgR protein. An overview of the ArgR–arginine complex is shown at the top of the image. The binding pocket of the wild type is on the bottom left, and the mutated strain is on the bottom right; the green stick represents the L-arginine molecule. **(B)** Relative transcription level of the reporter gene *sacB* in the mutant strains S127I, D146E, and D146L at an L-arginine concentration of 60 mM.

Since strains with high L-arginine production could reach growth limitation on 10% sucrose plates, the sensitivity of the ArgR protein to L-arginine was decreased so that the ArgR protein could only be activated by higher L-arginine concentrations. First, the amino acid sequence of ArgR was submitted to SWISS-MODEL to predict the structure model (Figure 6A). The crystal structure of the protein (PDB: 6A2Q) was used as a template for modeling, and 171 residues (53.8% of the sequence) were modeled using the single highest scoring template.

The molecular structure of ArgR was introduced to search for a mutation site that could cause ArgR to be insensitive to L-arginine. Residues S127 and D146 within the binding pocket were replaced by other amino acids. Finally, RT-qPCR was used to analyze the *sacB* expression in the site-saturation libraries. When 60 mM of L-arginine was added, some mutants showed changes in sensitivity. S127 was replaced by L-isoleucine, and D146 was replaced by L-glutamate and L-leucine. The expression of *sacB* was decreased by 2.38- and 1.27-fold for the D146E and D146L mutants, respectively, compared to the wild type, and expression was increased by 2.26-fold for the S127I mutant (Figure 6B). Therefore, insensitivity of ArgR to L-arginine was achieved by the mutant of S127I. The molecular structure simulation of S127I showed that the binding pocket of ArgR to L-arginine was diminished, which could increase the L-arginine screening limitation and improve the screening efficiency.

### Improvement of L-Arginine Production Based on the *sacB* Reporter Gene

**FIGURE 7:**
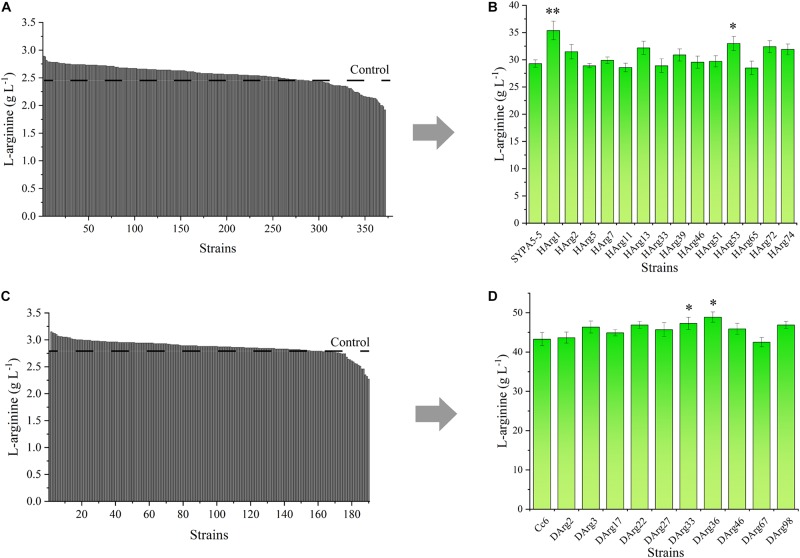
**(A)** L-Arginine yield of 372 larger mutant colonies of *C. crenatum* SYPA5-5 in 24-deep-well plates. **(B)** L-Arginine yield of the top 10% of mutant strains of *C. crenatum* SYPA5-5 in shake-flask fermentation. Statistically significantly increased L-arginine production are marked with asterisks, which were calculated by Student’s unpaired *t*-test (^∗^*P* ≤ 0.05; ^∗∗^*P* ≤ 0.01; ^∗∗∗^ *P* ≤ 0.001). **(C)** L-Arginine yield of 189 larger mutant colonies of Cc6 in 24-deep-well plates. **(D)** L-Arginine yield of the top 10% of mutant strains of Cc6 in shake-flask fermentation. Statistically significantly increased L-arginine production are marked with asterisks, which were calculated by Student’s unpaired *t*-test (^∗^*P* ≤ 0.05; ^∗∗^*P* ≤ 0.01).

For the L-arginine high-producing strain Cc6, the ARG-Select biosensor could be used to screen increased L-arginine producers. Based on the pSenArg-*sacB* biosensor plasmid, the *C. crenatum* SYPA5-5 and Cc6 strains were treated with ARTP mutagenesis to increase the L-arginine production. Mutagen-treated cells of the two strains were cultivated on preliminary screening plates for 48 h. For *C. crenatum* SYPA5-5, approximately 1,994 colonies grew on the plates, which were selected from the original 100,000 mutant cells. Because of the relationship between cell growth and L-arginine production, 18.7% of larger colonies were picked, and consequently, 372 large colonies were cultivated in 24-deep-well plates for 48 h (Figure 7A). The Sakaguchi reagent spectrophotometric method was used for L-arginine determination. Then, 14 mutant strains showed 10% increased L-arginine production, and the HArg1 strain could produce 2.89 g L^–1^ of L-arginine. The L-arginine yields of shake-flask fermentation of the 14 mutant strains were further confirmed by HPLC (Figure 7B). The HArg1 strain showed a stable L-arginine yield of 35.9 ± 2.0 g L^–1^, which represented a 20.8% improvement.

Similarly, for the Cc6 strain, approximately 1,852 colonies grew on sucrose plates, and only 189 large colonies were cultivated in 24-deep-well plates (Figure 7C); 10 strains exhibited a 10% increase in L-arginine production. For the shake-flask fermentation of the Cc6 strain, the DArg36 strain showed the highest L-arginine production, which was increased by 12.9%, and could produce 47.9 ± 1.7 g L^–1^ of L-arginine (Figure 7D).

### Fed-Batch Fermentation of the HArg1 and DArg36 Strains

**FIGURE 8:**
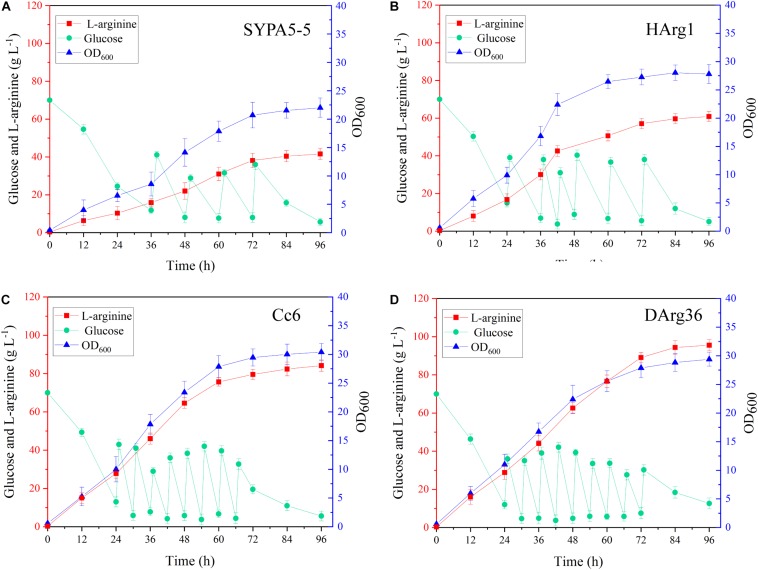
**(A)** Fed-batch fermentation of *C. crenatum* SYPA5-5. **(B)** Fed-batch fermentation of the HArg1 strain. **(C)** Fed-batch fermentation of Cc6. **(D)** Fed-batch fermentation of the DArg36 strain. Symbols denote: L-arginine (red squares), glucose (green circles), and OD_600_ (blue triangles).

The L-arginine production performances of the HArg1 and DArg36 strains were further investigated in 5-L bioreactors. Fed-batch cultures were performed to examine the performance of the mutant strains for L-arginine production over 96 h of fermentation. As shown in Figure 8, for the HArg1 strain, cell growth increased to an OD_600_ of 27.8 from 22.3 within 96 h. Because no sucrose was added, the cell growth of DArg36 was OD_600_ = 29.4, similar to the original Cc6 strain OD_600_ = 30.3. L-Arginine production of both the HArg1 and DArg36 strains was improved, and the glucose consumption was increased. The L-arginine production of HArg1 was 60.7 g L^–1^, which was increased by 44.5%, the productivity was 0.63 g L^–1^ h^–1^, the glucose consumption was 239.9 g L^–1^, and the L-arginine yield on glucose was 0.253 g g^–1^. The L-arginine production of the DArg36 strain was 95.5 g L^–1^, which was increased by 13.5%, the productivity was 0.99 g L^–1^ h^–1^, the glucose consumption was 348.7 g L^–1^, and the L-arginine yield on glucose was 0.273 g g^–1^. These results demonstrated that the ARG-Select biosensor was capable of obtaining hyperproducing strains with high efficiency.

## Discussion

Strains treated by mutagenesis can generate a large number of mutants. Screens and selections are the key components to obtain desired strains (Rogers et al., 2016). In recent years, an increasing focus has been placed on biosensors for high-throughput screening of hyperproducing strains; strains can be directly selected by the output of reporter gene. Here, we used an ARG-Select biosensor to screen high L-arginine producers of *C. crenatum* SYPA5-5, and this biosensor mainly consists of the repressor protein of ArgR and its corresponding *argC* promoter. Using *sacB* as the reporter gene, it can provide the strain with an output of bacterial growth in the presence of 10% sucrose. Therefore, the ARG-Select biosensor could translate the intracellular product level into a detectable output of bacterial growth. Because high L-arginine producing strains could reach the growth limitation on sucrose plates, site-saturation mutagenesis was applied to ArgR to decrease the sensitivity to L-arginine. In the ARG-Select biosensor system, negative mutant strains cannot survive under sucrose pressure, and colonies with sizes larger than the parent strain were used to measure the L-arginine production, greatly reducing the workload by a simple manipulation.

Previously, metabolic engineering strategies were used to balance enzyme expression levels and optimize genes encoding key enzymes (Brockman and Prather, 2015; Venayak et al., 2015). In *C. glutamicum* sp., a series of enzymes are involved in the L-arginine synthesis pathway. The expression of these enzymes is closely related to L-arginine production. For example, the overexpression of the *arg* cluster in *C. crenatum* SYPA5-5 by its native promoter can increase the L-arginine yield by approximately 24.9% (Xu et al., 2011b). The co-expression of the *argGH* transcript in *C. crenatum* SYPA5-5 can improve L-arginine production by 9.5% (Zhao et al., 2016).

However, the limitation of these enzymes makes it difficult for this strategy to achieve a higher level of metabolite production (Suter and Rosenbusch, 1977). Therefore, the strategy of random mutagenesis is still a desired approach that has a greater possibility of obtaining high-level production strains (Dietrich et al., 2010; Rogers et al., 2016), and biosensors have emerged as a high-throughput screening method to obtain hyperproducing strains. The biosensors are mainly based on recognition elements that can respond to specific metabolites, such as TFs (Mannan et al., 2017; Hanko et al., 2018), riboswitches (Blouin et al., 2009), fluorescence resonance energy transfer (FRET) (Zhang et al., 2011), and pressure-sensitive promoters (Xie et al., 2015). Among these elements, TFs play a major role in controlling gene expression at the level of transcription, and TFs-based biosensors have been widely applied to obtain high-yield strains by monitoring intracellular metabolite production from a measurable output (Cheng et al., 2018).

In this study, the increased L-arginine production of *C. crenatum* SYPA5-5 and Cc6 strains demonstrated the effectiveness of the biosensor system. This is a valuable high-throughput screening method that can bridge the gap between a detectable signal and metabolite production. Furthermore, we believe that the L-arginine production of our strains still has an improvement space. Adaptive laboratory evolution (ALE) will be applied to increase the L-arginine production, which is achieved by further mutation and screening.

## Data Availability Statement

All datasets generated for this study are included in the article/supplementary material.

## Author Contributions

MX and PL performed the experiments and wrote the manuscript. JC and AP helped in the analysis design and data interpretation. ZR designed the experiments and reviewed the manuscript. All authors reviewed the manuscript.

## Conflict of Interest

The authors declare that the research was conducted in the absence of any commercial or financial relationships that could be construed as a potential conflict of interest.

## Footnotes

1 https://swissmodel.expasy.org/

## References

[B1] Alonso S. Rendueles M. Diaz M. (2015). Microbial production of specialty organic acids from renewable and waste materials. *Crit. Rev. Biotechnol.* 35 497–513. 10.3109/07388551.2014.904269 24754448

[B2] Ami P. Nihir S. Prajapati J. B. (2013). Biosynthesis of vitamins and enzymes in fermented foods by lactic acid bacteria and related genera – a promising approach. *Croat. J. Food Sci. Technol.* 5 85–91. 10.1016/S0009-2797(02)00067-4

[B3] Blouin S. Mulhbacher J. Penedo J. C. Lafontaine D. A. (2009). Riboswitches: ancient and promising genetic regulators. *Chembiochem* 10 400–416. 10.1002/cbic.200800593 19101979

[B4] Brockman I. M. Prather K. L. (2015). Dynamic metabolic engineering: new strategies for developing responsive cell factories. *Biotechnol. J.* 10 1360–1369. 10.1002/biot.201400422 25868062 PMC4629492

[B5] Chen X. L. Zhang B. Tang L. Jiao H. T. Xu H. Y. Xu F. (2014). Expression and characterization of ArgR, an arginine regulatory protein in *Corynebacterium crenatum*. *Biomed. Environ. Sci.* 27 436–443. 10.3967/bes2014.072 24961853

[B6] Cheng F. Tang X. L. Kardashliev T. (2018). Transcription factor-based biosensors in high-throughput screening: advances and applications. *Biotechnol. J.* 13:e1700648. 10.1002/biot.201700648 29485214

[B7] Dietrich J. A. Mckee A. E. Keasling J. D. (2010). High-throughput metabolic engineering: advances in small-molecule screening and selection. *Annu. Rev. Biochem.* 79 563–590. 10.1146/annurev-biochem-062608-095938 20367033

[B8] Gay P. Coq D. Le Steinmetz M. Ferrari E. Hoch J. A. (1983). Cloning structural gene sacB, which codes for exoenzyme levansucrase of *Bacillus subtilis*: expression of the gene in *Escherichia coli*. *J. Bacteriol.* 153 1424–1431. 6402497 10.1128/jb.153.3.1424-1431.1983 PMC221793

[B9] Grell L. Parkin C. Slatest L. Biochemistry P. A. C. J. (2006). EZ-Viz, a tool for simplifying molecular viewing in PyMOL. *Biochem. Mol. Biol. Educ.* 34 402–407. 10.1002/bmb.2006.494034062672 21638731

[B10] Guo J. Man Z. Rao Z. Xu M. Yang T. Zhang X. (2017). Improvement of the ammonia assimilation for enhancing L-arginine production of *Corynebacterium crenatum*. *J. Ind. Microbiol. Biotechnol.* 44 443–451. 10.1007/s10295-017-1900-9 28120129

[B11] Hagelueken G. Ward R. Naismith J. H. Resonance O. S. J. A. M. (2012). MtsslWizard: in silico spin-labeling and generation of distance distributions in PyMOL. *Appl. Magn. Reson.* 42 377–391. 10.1007/s00723-012-0314-0 22448103 PMC3296949

[B12] Hanko E. K. R. Minton N. P. Malys N. (2018). A transcription factor-based biosensor for detection of itaconic acid. *ACS Synth. Biol.* 7 1436–1446. 10.1021/acssynbio.8b00057 29638114 PMC6345495

[B13] Kubicek C. P. Mikus M. Schuster A. Schmoll M. Seiboth B. (2009). Metabolic engineering strategies for the improvement of cellulase production by *Hypocrea jecorina*. *Biotechnol. Biofuels* 2:19. 10.1186/1754-6834-2-19 19723296 PMC2749017

[B14] Liu Q. Kasuga M. Sakuma Y. Abe H. Miura S. Yamaguchi-Shinozaki K. (1998). Two transcription factors, DREB1 and DREB2, with an EREBP/AP2 DNA binding domain separate two cellular signal transduction pathways in drought- and low-temperature-responsive gene expression, respectively, in *Arabidopsis*. *Plant Cell* 10 1391–1406. 10.1105/tpc.10.8.1391 9707537 PMC144379

[B15] Liu Y. Zhuang Y. Ding D. Xu Y. Sun J. Zhang D. (2017). Biosensor-based evolution and elucidation of a biosynthetic pathway in *Escherichia coli*. *ACS Synth. Biol.* 6 837–848. 10.1021/acssynbio.6b00328 28121425

[B16] Mahr R. Gatgens C. Gatgens J. Polen T. Kalinowski J. Frunzke J. (2015). Biosensor-driven adaptive laboratory evolution of l-valine production in *Corynebacterium glutamicum*. *Metab. Eng.* 32 184–194. 10.1016/j.ymben.2015.09.017 26453945

[B17] Man Z. Xu M. Rao Z. Guo J. Yang T. Zhang X. (2016). Systems pathway engineering of *Corynebacterium crenatum* for improved L-arginine production. *Sci. Rep.* 6:28629. 10.1038/srep28629 27338253 PMC4919616

[B18] Mannan A. A. Liu D. Zhang F. Oyarzun D. A. (2017). Fundamental design principles for transcription-factor-based metabolite biosensors. *ACS Synth. Biol.* 6 1851–1859. 10.1021/acssynbio.7b00172 28763198

[B19] Messineo L. (1966). Modification of the Sakaguchi reaction: spectrophotometric determination of arginine in proteins without previous hydrolysis. *Arch. Biochem. Biophys.* 117 534–540. 10.1016/0003-9861(66)90094-4

[B20] Mitić S. S. Miletić G. Ž Pavlović A. N. Tošić S. B. Velimirović D. S. (2013). Development and Evaluation of a kinetic−spectrophotometric method for determination of arginine. *J. Chin. Chem. Soc.* 54 47–54. 10.1002/jccs.200700009

[B21] Park S. H. Kim H. U. Kim T. Y. Park J. S. Kim S. S. Lee S. Y. (2014). Metabolic engineering of *Corynebacterium glutamicum* for L-arginine production. *Nat. Commun.* 5 45–54. 10.1038/ncomms5618 25091334

[B22] Pelicic V. Reyrat J. M. Gicquel B. (1996). Expression of the *Bacillus subtilis* sacB gene confers sucrose sensitivity on mycobacteria. *J. Bacteriol.* 178 1197–1199. 10.1128/jb.178.4.1197-1199.1996 8576057 PMC177784

[B23] Rogers J. K. Taylor N. D. Church G. M. (2016). Biosensor-based engineering of biosynthetic pathways. *Curr. Opin. Biotechnol.* 42 84–91. 10.1016/j.copbio.2016.03.005 26998575

[B24] Schendzielorz G. Dippong M. Grünberger A. Kohlheyer D. Yoshida A. Binder S. (2014). Taking control over control: use of product sensing in single cells to remove flux control at key enzymes in biosynthesis pathways. *ACS Synth. Biol.* 3 21–29. 10.1021/sb400059y 23829416

[B25] Schneider J. Niermann K. Wendisch V. F. (2011). Production of the amino acids l-glutamate, l-lysine, l-ornithine and l-arginine from arabinose by recombinant *Corynebacterium glutamicum*. *J. Biotechnol.* 154 191–198. 10.1016/j.jbiotec.2010.07.009 20638422

[B26] Scognamiglio V. Antonacci A. Lambreva M. D. Litescu S. C. Rea G. (2015). Synthetic biology and biomimetic chemistry as converging technologies fostering a new generation of smart biosensors. *Biosens. Bioelectron.* 74 1076–1086. 10.1016/j.bios.2015.07.078 26277908

[B27] Siedler S. Stahlhut S. G. Malla S. Maury J. Neves A. R. (2014). Novel biosensors based on flavonoid-responsive transcriptional regulators introduced into *Escherichia coli*. *Metab. Eng.* 21 2–8. 10.1016/j.ymben.2013.10.011 24188962

[B28] Sun D. S. Wang H. J. Zhang Q. (2013). Breeding of *Bacillus mucilaginosus* HJ07 mutated by UV/NTG and its efects on microbial leaching of bauxite. *J. Univ. Sci. Technol. Beijing* 35 1269–1278.

[B29] Suter P. Rosenbusch J. P. (1977). Asymmetry of binding and physical assignments of CTP and ATP sites in aspartate transcarbamoylase. *J. Biol. Chem.* 252 8136–8141. 10.1016/0020-711X(77)90060-X 334776

[B30] Theron G. Reid S. J. (2011). ArgR-promoter interactions in *Corynebacterium glutamicum* arginine biosynthesis. *Biotechnol. Appl. Biochem.* 58 119–127. 10.1002/bab.15

[B31] Venayak N. Anesiadis N. Cluett W. R. Mahadevan R. (2015). Engineering metabolism through dynamic control. *Curr. Opin. Biotechnol.* 34 142–152. 10.1016/j.copbio.2014.12.022 25616051

[B32] Wendisch V. F. (2014). Microbial production of amino acids and derived chemicals: synthetic biology approaches to strain development. *Curr. Opin. Biotechnol.* 30 51–58. 10.1016/j.copbio.2014.05.004 24922334

[B33] Xie W. Ye L. Lv X. Xu H. Yu H. (2015). Sequential control of biosynthetic pathways for balanced utilization of metabolic intermediates in *Saccharomyces cerevisiae*. *Metab. Eng.* 28 8–18. 10.1016/j.ymben.2014.11.007 25475893

[B34] Xu D. Tan Y. Shi F. Wang X. (2010). An improved shuttle vector constructed for metabolic engineering research in *Corynebacterium glutamicum*. *Plasmid* 64 85–91. 10.1016/j.plasmid.2010.05.004 20580910

[B35] Xu M. Rao Z. Dou W. Xu Z. (2013). The role of ARGR repressor regulation on L-arginine production in *Corynebacterium crenatum*. *Appl. Biochem. Biotechnol.* 170 587–597. 10.1007/s12010-013-0212-4 23564434

[B36] Xu M. Rao Z. Xu H. Lan C. Dou W. Zhang X. (2011a). Enhanced production of L-arginine by expression of *Vitreoscilla* hemoglobin using a novel expression system in *Corynebacterium crenatum*. *Appl. Biochem. Biotechnol.* 163 707–719. 10.1007/s12010-010-9076-z 20835781

[B37] Xu M. Rao Z. Yang J. Xia H. Dou W. Jin J. (2011b). Heterologous and homologous expression of the arginine biosynthetic *arg*C~H cluster from *Corynebacterium crenatum* for improvement of L-arginine production. *J. Ind. Microbiol. Biotechnol.* 39 495–502. 10.1007/s10295-011-1042-4 22009057

[B38] Yakovleva L. Chen S. Hecht S. M. Shuman S. (2008). Chemical and traditional mutagenesis of vaccinia DNA topoisomerase provides insights to cleavage site recognition and transesterification chemistry. *J. Biol. Chem.* 283 16093–16103. 10.1074/jbc.M801595200 18367446 PMC2414267

[B39] Yim S. H. Jung S. Lee S. K. Cheon C. I. Song E. Lee S. S. (2011). Purification and characterization of an arginine regulatory protein, ArgR, in *Corynebacterium glutamicum*. *J. Ind. Microbiol. Biotechnol.* 38 1911–1920. 10.1007/s10295-011-0977-9 21559975

[B40] Zhang C. Yuan Y. Zhang S. Wang Y. Liu Z. (2011). Biosensing platform based on fluorescence resonance energy transfer from upconverting nanocrystals to graphene oxide. *Angew. Chem. Int. Ed. Engl.* 50 6851–6854. 10.1002/anie.201100769 21656878

[B41] Zhang F. Carothers J. M. Keasling J. D. (2012). Design of a dynamic sensor-regulator system for production of chemicals and fuels derived from fatty acids. *Nat. Biotechnol.* 30 354–359. 10.1038/nbt.2149 22446695

[B42] Zhao Q. Luo Y. Dou W. Zhang X. Zhang X. Zhang W. (2016). Controlling the transcription levels of argGH redistributed L-arginine metabolic flux in N-acetylglutamate kinase and ArgR-deregulated *Corynebacterium crenatum*. *J. Ind. Microbiol. Biotechnol.* 43 55–66. 10.1007/s10295-015-1692-8 26521658

